# Reduction of radiation exposure to operating physician and assistant using a real-time auditory feedback dosimeter during femoral artery puncturing: a study on swine model

**DOI:** 10.1186/s41747-019-0116-3

**Published:** 2019-09-23

**Authors:** Muhammad Umair Ahmad Khan, Byung-Ju Yi

**Affiliations:** 1 Department of Mechatronics Engineering, Hanyang University, Ansan, Gyeonggi-do South Korea; 2 Department of Electronic Systems Engineering, Hanyang University, 55 Hanyangdeahak-ro, Sangnok-gu, Ansan, Gyeonggi-do 15588 South Korea

**Keywords:** Fluoroscopy, Occupational exposure, Radiation exposure, Radiation protection, Radiometry

## Abstract

**Background:**

Real-time dosimeters may create a relatively safer environment not only for the patient but also for the physician and the assistant as well. We propose the use of a real-time radiation measurement dosimeter having auditory feedback to reduce radiation exposure.

**Methods:**

Radiation dose rates were measured for 30 fluoroscopy-guided puncturing procedures of femoral arteries in swine. Fifteen puncturing procedures were performed with real-time radiation measurement dosimeter having auditory feedback and other 15 were performed without auditory feedback dosimeter by an interventional cardiologist with 10 years of experience.

**Results:**

The left body side of the operating physician (38%, *p* < 0.001) and assistant (25%, *p* < 0.001) was more exposed as compared to the right body side. Radiation dose rate to the left hand, left arm and left leg were reduced from 0.96 ± 0.10 to 0.79 ± 0.12 mSv/h (17% reduction, *p* < 0.001), from 0.11 ± 0.02 to 0.07 ± 0.01 mSv/h (36% reduction, *p* < 0.001) and from 0.22 ± 0.06 to 0.15 ± 0.02 mSv/h (31% reduction, *p* < 0.001) with the use of auditory feedback dosimeter, respectively. The mean fluoroscopic time was reduced from 4.8 ± 0.43 min to 4.2 ± 0.53 min (*p* < 0.001). The success rate of performing arterial puncturing was 100%.

**Conclusions:**

The use of auditory feedback dosimeter resulted in reduction in effective dose. The sound beep alerted the physician from the danger of exposure, and this approach induced awareness and protective mindset to the operating physician and assistant.

## Key points

- Fluoroscopy-guided puncturing of femoral arteries in pigs is performed with (*n* = 15) and without (*n* = 15) a real-time auditory feedback dosimeter
- Radiation dose rate to the left hand of the operating physician was significantly reduced from 0.96 to 0.79 mSv/h (−17%).
- Radiation dose rate to the left arm of the operating physician was significantly reduced from 0.11 to 0.07 mSv/h (−36%).
- Radiation dose rate to the left leg of the operating physician was significantly reduced from 0.22 to 0.15 mSv/h (−31%).
- Fluoroscopy time was significantly reduced from 4.8 to 4.2 min.

## Background

Fluoroscopy is commonly used for precisely targeting and placing needles and guidewires during interventional procedures. However, it results in radiation exposure for the operating physician (OP), the patient and the medical personnel [1–6]. The continuous exposure of radiation will cause severe damages of biological nature for humans exposed [7, 8].

Complex vascular interventional procedures involve radiation exposure for the OP, patients and the medical staff assisting the physicians [9]. The effective dose rate varies from *μ*Sv to mSv per procedure. Radiation dose to the OP’s hands had been measured in various studies [10–12]. The average value of eye lens dose limit has been reduced to 20 mSv per year for 5 years, without exceeding 50 mSv in a single year [13].

Various techniques have been proposed to reduce the radiation exposure in the operating room, mainly by optimisation of fluoroscopic devices, sometimes reducing the image quality. Of note, proper positioning of x-ray source and operating fluoroscopic devices in pulsed mode can significantly reduce radiation [14–16]. Protective shielding is another way to reduce the radiation dose to the medical staff. It includes thyroid collars and lead aprons used as a protection against radiation exposure [17].

The main limitation of thermoluminescence dosimeters is given by the availability of exposure data only at a later stage [16, 18]. Conversely, real-time radiation dosimetry provides radiation exposure feedback in real-time so that the OP and whole staff can prevent increased exposure by changing position/orientation of the fluoroscopic devices and maintaining a safe distance. Radiation awareness among the medical staff and physicians can be augmented by giving them personalised real-time feedback about the radiation dose, changing the behaviour of the medical staff [19].

In addition, current radiation measurement techniques provide cumulative dose and real-time radiation measurement devices have been proposed but they have the great limitation of being highly expensive [20].

In this study, we propose a cheap and portable radiation measurement technique using real-time radiation measurement dosimeter having auditory feedback.

## Methods

This study was approved by the Animal Ethical Committee of Seoul National University Bundang Hospital (code of approval: BA1708-230/075-01). The procedures were performed in accordance with the Guide for the Care and Use of Laboratory Animals from the Institute of Laboratory Animals Resources [21].

### Animal preparation

The day before the experiment, male crossbred swine (*n* = 15; weight, from 17 to 35 kg) were fed with aspirin (300 mg) and clopidogrel (300 mg). On the experiment day, the swine were premedicated with atropine sulfate (0.05 mg/kg, intramuscularly) and subsequently anaesthetised with Zoletil (5 mg/kg) and Xylazine (4.4 mg/kg) intramuscularly, intubated and ventilated with room air and isoflurane. We inserted a 6-Fr sheath *via* the right carotid artery by ultrasound-guided puncture. The animals received heparin (5000 U) intravenously prior to femoral artery digital subtraction angiography.

### Real-time radiation measuring device

**Fig. 1:**
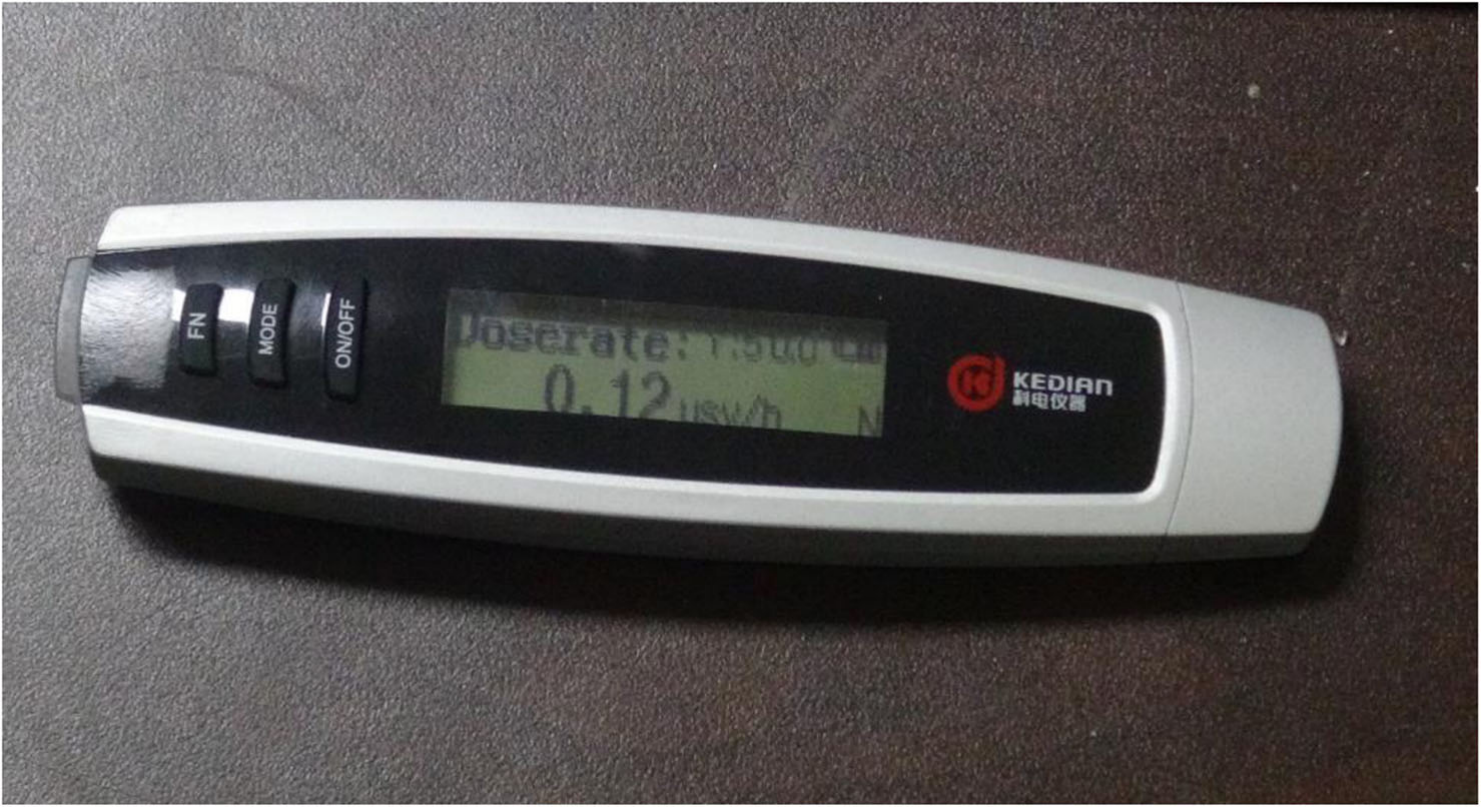
X-ray dosimeter Ray 3000. It measures the radiation dose rates in millisievert per hour and microsievert per hour

A real-time radiation measuring dosimeter (Ray 3000, Kedian, Jining, China) with auditory feedback was used to measure the radiation dose rates (Fig. 1). It can measure the radiation dose rate ranges from 0.01 *μ*Sv/h to 99.99 mSv/h. It has five threshold alarm settings (0.5, 1, 2.5, 10, 30, 50 *μ*Sv/h). Alarm type can be set either pulsed, continuous or silent. The device was set at 10 *μ*Sv/h as the threshold value.

### Fluoroscopy-guided arterial puncture

A total of 30 fluoroscopy-guided punctures were performed, 15 using the real-time radiation measurement dosimeter with auditory feedback and 15 without auditory feedback dosimeter (the dosimeter was set in silent mode) by an interventional cardiologist with 10-year experience, targeting both femoral arteries in the 15 pigs. The mobile fluoroscopy system was Philips BV Pulsera (Philips Medical Systems, Bothell, USA). We selected both femoral arteries with a diagnostic catheter (Judkins right 4, Gifu, Japan) *via* the right carotid artery and performed angiography to guide the femoral puncture using the Seldinger technique.

### Measurement of radiation dose and fluoroscopic time

**Fig. 2:**
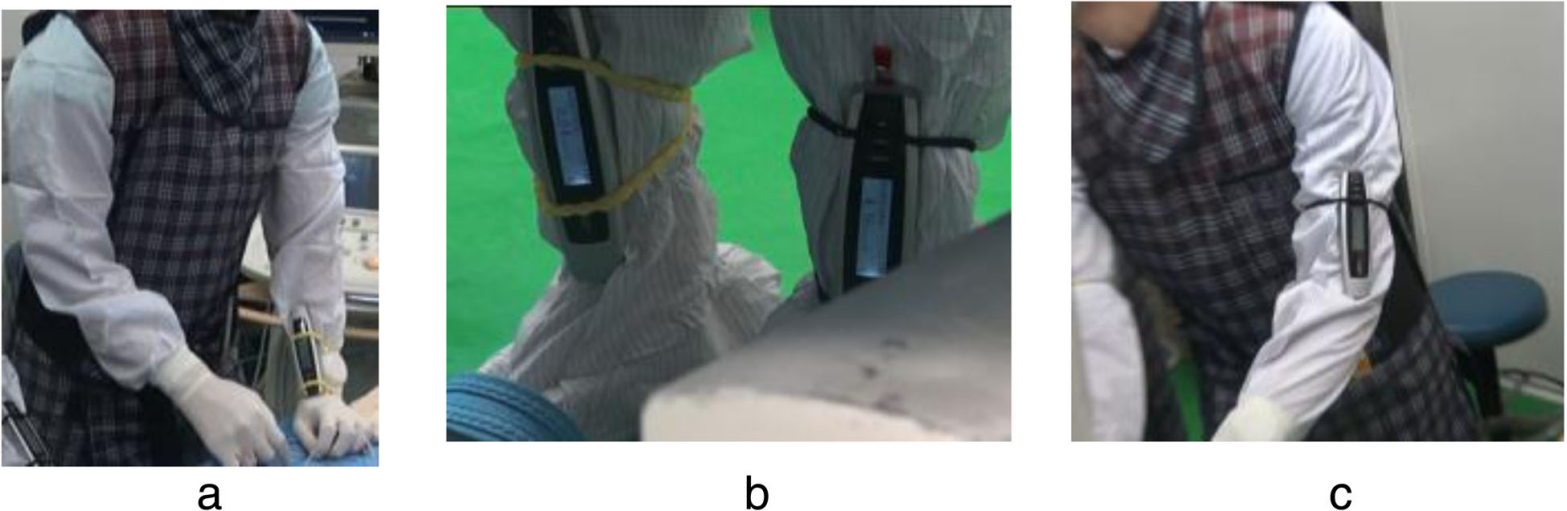
Location of dosimeter for the operating physician. The dosimeter is attached to various unprotected body parts of the operating physician to measure radiation dose rates: **a** hands, **b** legs, **c** arms

**Fig. 3:**
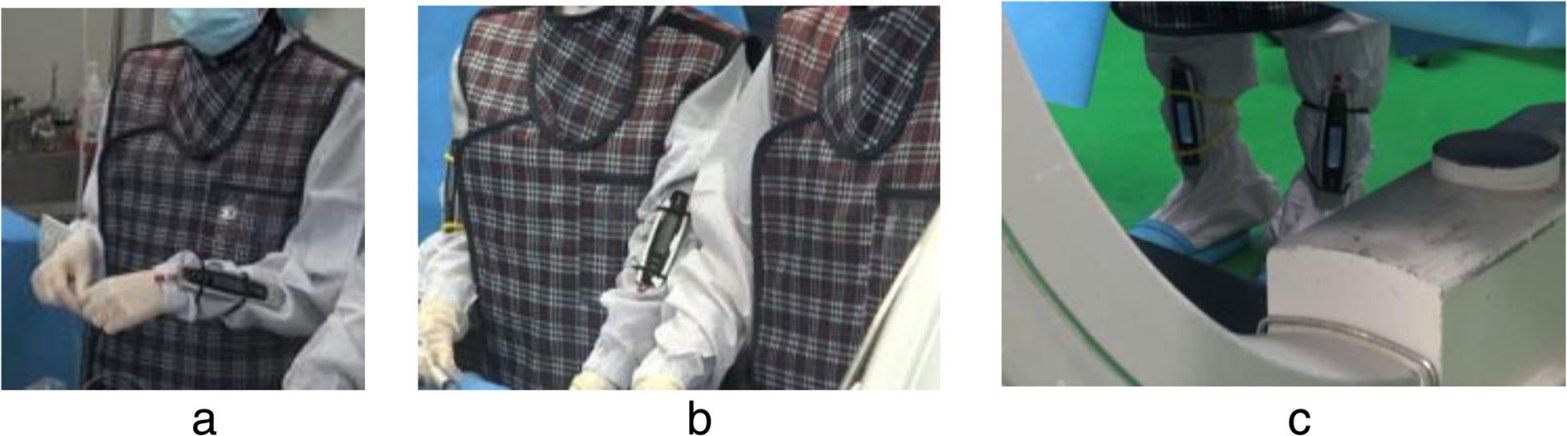
Location of dosimeter for the assistant. The dosimeter is attached to various unprotected body parts of the assistant to measure radiation dose rates: **a** hands, **b** arms, **c** legs

In each case, the OP wore the dosimeters at three different positions, attached to hands, arms, and legs of the physician and the assistant in order to measure the radiation dose rate as shown in Fig. 2 and Fig. 3, respectively. All the dose rates were measured in millisievert per hour. Mean fluoroscopic time had also been measured with and without auditory feedback dosimeter.

### Position of physician and C-arm in the operating theatre

In clinical practice, when performing fluoroscopy, two positions of the C-arm are commonly adopted. In these positions, either left or right body side of the OP is more exposed as compared to the other. In this study, the C-arm was positioned such that the left body side of the OP and the assistant was more exposed as compared to the right body side. The distance between the unprotected parts of OP and the swine varied depending upon the position and orientation of the OP. The hands, legs, and arms were the unprotected parts of the body and the distance was not constant, varying as the OP changes the position. However, the range of the arm distance from the swine was about 20 to 30 cm and the range of the leg distance from the swine was about 40 to 50 cm. The distance between the x-ray source and the swine was about 80 cm and the distance between the intensifier and the swine was about 30 cm.

### Statistical analysis

Continuous variables were reported as mean ± standard deviation, taking into consideration their normal or near-normal distribution, confirmed by Shapiro-Wilk test (*p =* 0.880). As a consequence, the comparison between the means of the two groups was evaluated by Student *t* test. A two-sided probability value of < 0.05 was considered indicative of a statistically significant difference. The success rates were presented as percentages with their 95% confidence intervals, calculated according to the binomial distribution. Statistical analysis was performed using R programme version 3.1.0 (The R Foundation, Vienna, Austria).

## Results

The success rate of performing arterial puncturing was 15/15 (100%, 95% confidence interval 0.78–1.00), both with or and without auditory feedback dosimeter. Table 1 shows the radiation dose rate for the OP and the assistant. The mean values of radiation dose rates for the left and right hand were 0.96 mSv/h and 0.74 mSv/h, respectively. Similarly, the mean values of radiation dose rates for the left arm and left leg were 0.11 mSv/h and 0.35 mSv/h, respectively.

**Table 1:** Radiation dose rate (mSv/h) for the operating physician and the assistant

	Left hand (*n* = 15)	Right hand (*n* = 15)	Left leg (*n* = 15)	Right leg (*n* = 15)	Left arm (*n* = 15)	Right arm (*n* = 15)
Dose rate for the operating physician (mean ± SD)	0.960 ± 0.100	0.740 ± 0.190	0.220 ± 0.060	0.140 ± 0.030	0.110 ± 0.020	0.060 ± 0.010
*p*-value	< 0.001		< 0.001		< 0.001	
Dose rate for the assistant (mean ± SD)	0.090 ± 0.010	0.070 ± 0.010	0.020 ± 0.010	0.010 ± 0.004	0.030 ± 0.010	0.010 ± 0.003
*p*-value	< 0.001		< 0.001		< 0.001	

*SD* Standard deviation

The radiation exposure of assistant was significantly (−90%, *p* < 0.001) lower as compared to the OP. The left side of the assistant was more exposed as compared to the right side. The mean values of radiation dose rates for the left and right hand were 0.10 mSv/h and 0.06 mSv/h, respectively. Similarly, the mean values of radiation dose rates for the left arm and left leg were 0.05 mSv/h and 0.04 mSv/h, respectively.

Table 2 shows the mean fluoroscopic exposure time and radiation dose rate for the left hand, arm and leg with and without using dosimeter having auditory feedback. It was reduced from 4.8 to 4.2 min with the use of auditory feedback dosimeter. The mean value of radiation dose rate to left hand, left arm and left leg had been reduced from 0.96 ± 0.10 to 0.79 ± 0.12 mSv/h, 0.11 ± 0.02 mSv/h without the use of auditory feedback dosimeter to 0.07 ± 0.01 mSv/h and 0.22 ± 0.06 to 0.15 ± 0.02, respectively, with the use of auditory feedback dosimeter.

**Table 2:** Fluoroscopic exposure time and radiation dose rates with and without auditory feedback dosimeter

Parameters	Without auditory feedback dosimeter (*n* = 15)	With auditory feedback dosimeter (*n* = 15)
Fluoroscopic exposure time (min) (mean ± standard deviation)	4.800 ± 0.430	4.200 ± 0.530
*p*-value	< 0.001	
Radiation dose rate for the left hand (mSv/h)	0.960 ± 0.100	0.790 ± 0.120
*p*-value	< 0.001	
Radiation dose rate for the left arm (mSv/h)	0.110 ± 0.020	0.070 ± 0.010
*p*-value	< 0.001	
Radiation dose rate for the left leg (mSv/h)	0.220 ± 0.060	0.150 ± 0.020
*p*-value	< 0.001	

## Discussion

The objective of the study was the use of real-time radiation dosimeter having auditory feedback to reduce radiation exposure for both the OP and the assistant. It was found that the OP was significantly more exposed as compared to the assistant. It was also observed that the left body side of the OP and assistant was significantly more exposed as compared to the right one. With the use of real-time radiation dosimeter having auditory feedback, 17% decrease in radiation exposure for the left hand, 36% decrease for the left arm and 31% decrease for the left leg was observed. The mean fluoroscopy time was slightly by significantly reduced.

Notably, the real-time radiation measurement dosimeter Ray 3000 with auditory feedback is not only cheap but also portable, being easily attachable to various body parts. The beep sound warning system based on auditory feedback allowed the operator and assistant to modify their behaviour to reduce radiation exposure and this approach helped in creating awareness among themselves. The use of the auditory feedback dosimeter allowed the OP to perform arterial puncturing with less exposure. In fact, when the OP and assistant did not wear dosimeters, they moved closer to the x-ray source in order to properly utilise the workspace. As a result, their bodies were more exposed to radiation. Of note, the effective dose increases with the increase in the weight of the patient [22] and studies have shown that for every 1 cm of thickness of body tissue, there is a 25% increase in the radiation exposure for the patient [23].

This technique could help in the reduction of radiation but still had limitations because both the OP and assistant work in close vicinity of the radiation environment. The use of robotic arterial puncturing mechanisms would allow the OP to perform procedures far from the radiation source so that the OP and assistant would be less exposed to scattered radiation [24]. Such dosimeters having auditory feedback would be used for the extravascular procedure where hand-body exposure is higher than vascular intervention. The other limitation of this study was a small sample size. However, the reduction of radiation for the left body side using auditory feedback dosimeter was significant.

In peripheral intervention, various puncture sites are required other than the femoral or radial artery, such as dorsalis pedis artery, posterior tibial artery, peroneal artery, or even plantar arteries. In those cases, it is hard to identify small arteries using sonography and it is much easier under the guidance of fluoroscopy and contrast angiography. The reason for more time consumption in arterial puncturing was the complications related to smaller arterial size in animal models and higher susceptibility of the arterial wall to complications. In the presence of human patients, real-time radiation dosimeter technique would be effective since the dosimeter has three alarm settings: pulsed, continuous and silent. In both pulsed and continuous case, the sound of the alarm is low as compared to the sound beep of the switching on of the fluoroscopic C-arm so this dosimeter would not disturb the patient.

In conclusion, the use of real-time radiation measurement dosimeter having auditory feedback had created awareness about the risk of exposure and significantly reduced radiation dose rates for the hands, arms and legs. The use of this technique would eventually lead us towards a safer environment not only for the patient but also for the OP and the medical staff.

## Data Availability

Individual measurement data are available on reasonable request.

## Abbreviations

OP: Operating physician
